# Labelling and Clinical Performance of Human Leukocytes Labelled with ^99m^Tc-HMPAO Using Leukokit® with Gelofusine versus Leukokit® with HES as Sedimentation Agent

**DOI:** 10.1155/2019/4368342

**Published:** 2019-03-25

**Authors:** S. Auletta, D. Riolo, M. Varani, C. Lauri, F. Galli, A. Signore

**Affiliations:** ^1^Nuclear Medicine Unit, Department of Medical-Surgical Sciences and of Translational Medicine, Faculty of Medicine and Psychology, “Sapienza” University of Rome, Rome, Italy; ^2^Department of Nuclear Medicine and Molecular Imaging, University Medical Center Groningen, University of Groningen, Groningen, Netherlands

## Abstract

The scintigraphy with radiolabelled autologous leukocytes (WBCs) is considered the gold-standard technique for imaging infections. Leukokit® is a commercially available, disposable, sterile kit for labelling WBCs ex vivo. In this kit, WBCs isolation from red blood cells (RBCs) was performed using poly(O-2-hydroxyethyl)starch (HES) as the RBCs sedimentation agent. Due to its poor availability, HES has been recently replaced by Gelofusine as the RBC sedimentation agent. The aim of this study was to compare the labelling efficiency and the diagnostic accuracy of WBCs labelled with Leukokit® with HES vs Leukokit® with Gelofusine. WBCs were isolated using HES or Gelofusine for 45 minutes and then purified from platelets (PLTs) and labelled with 1.1 ± 0.3 GBq of freshly prepared ^99m^Tc-HMPAO. The following parameters were evaluated: the number and type of recovered WBCs, RBCs contamination, PLTs contamination, vitality of neutrophils, and chemotactic properties of neutrophils. Clinical comparison was performed between 80 patients (33 males; age 67.5 ± 14.2) injected with ^99m^Tc-HMPAO-WBCs, using HES as the sedimentation agent, and 92 patients (38 males; age 68.2 ± 12.8) injected with ^99m^Tc-HMPAO-WBCs using Gelofusine as the sedimentation agent. Patients were affected by prosthetic joint infections, peripheral bone osteomyelitis, or vascular graft infection. We compared radiolabelling efficiency (LE), final recovery yield (RY), and diagnostic outcome based on microbiology or 2-year follow-up. Results showed that HES provides the lowest RBCs and PLTs contamination, but Gelofusine provides the highest WBC recovery. Both agents did not influence the chemotactic properties of WBCs, and no differences were found in terms of LE and RY. Sensitivity, specificity, and accuracy were also not significantly different for WBCs labelled with both agents (diagnostic accuracy 90.9%, CI = 74.9–96.1 vs 98.3%, CI = 90.8–100, for HES and Gelofusine, respectively). In conclusion, Gelofusine can be considered a suitable alternative of HES for WBCs separation and labelling.

## 1. Introduction

The scintigraphy with radiolabelled autologous leukocytes (WBCs) is considered the gold-standard technique for imaging infections, reaching a sensitivity and specificity between 95% and 100% according to site [1–3], even though several other agents are currently being developed for direct imaging of bacteria [4, 5]. WBCs are usually radiolabelled with two radiopharmaceuticals: ^99m^Tc-hexamethylpropylene amine oxime (^99m^Tc-HMPAO) or ^111^In-oxine, following the EANM guidelines [6, 7]. WBCs isolation from whole blood is a key procedure to obtain a pure and specific radiopharmaceutical and to perform radiolabelled leukocyte scintigraphy. Leukokit® (GI Pharma, Italy) is a commercially available, disposable, sterile kit for labelling WBCs ex vivo. In this kit, poly(O-2-hydroxyethyl)starch (HAES-steril 10%, HES) has been routinely used as a sedimentation agent to remove erythrocytes (RBCs) from WBCs [8–12]. However, HES is no longer commercially available, and it was replaced in Leukokit® with an alternative agent, Gelofusine (B. Braun, Germany).

The aim of the study is to test *in vitro* the suitability of Gelofusine as an alternative to HES. This was achieved through the assessment of several parameters after erythrocyte separation: the number and type of recovered WBCs; RBCs contamination; platelets (PLTs) contamination; viability of neutrophils; chemotactic properties of neutrophils.

After the evaluation of safety and efficacy of the new sedimentation agent (Gelofusine) performed by the producers of Leukokit® (GI Pharma, Italy), we aimed at evaluating the performance of this “new Leukokit®” (initially produced by GI Pharma, Italy, and now produced by CellTech, Italy), as compared to the previous kit containing HES (“old Leukokit®” produced by GI Pharma, Italy), for WBCs purification and labelling with ^99m^Tc-HMPAO, as requested by the Italian legislation.

The second goal of the study consists in the complete validation of the “new Leukokit®”, containing Gelofusine, as compared to the previously commercialized kit, containing HES, applying our standard operating procedure (SOP) for the validation and annual revalidation of the WBCs purification and labelling procedure.

Secondly, the “new” and the “old” Leukokit® were compared in terms of WBCs labelling efficiency, recovery yield, and diagnostic accuracy in patients with suspected infections.

## 2. Materials and Methods

### 2.1. WBC Isolation

For the *in vitro* study, WBCs were isolated from the blood of 5 healthy volunteers. In brief, 30 ml of blood was withdrawn from each subject with a syringe containing 6 ml of anticoagulant citrate dextrose (ACD). The blood was then divided in 3 Falcon-type tubes (12 ml each) containing 3 ml of HES, Gelofusine, or 0.9% NaCl solution, respectively. Gelofusine was provided at 4% concentration of a clear, transparent, and slightly yellowish sterile solution (catalogue no. 152117651, B. Braun, Germany). After approximately 40 minutes of sedimentation, cell-rich plasma (CRP) was collected from each vial and an aliquot was used for FACS analysis to evaluate the number of WBCs, RBCs, and PLTs contaminations. Another aliquot was used for the viability testing by the trypan blue exclusion test. The remaining CRP was then centrifuged on Lymphoprep® for 10 minutes at 1000 rpm to isolate granulocytes. After the centrifugation, the supernatant was discarded and the pellet was resuspended in 5 ml of phosphate buffered saline (PBS). FACS analysis and viability test were then repeated, and the rest was used to evaluate the retention of the migrating capabilities of granulocytes.

### 2.2. Migration Assay for Granulocytes

Granulocytes migration was evaluated using a 24-well permeable support with 5 *μ*m pores (Corning®) placed in a 24 multiwell plate. In the upper chamber of each well were placed 10^5^ granulocytes in 100 *μ*l of RPMI, whereas the lower chamber of each well contained 650 *μ*l of RPMI supplemented with 10% FBS. The plate was then incubated overnight in an incubator at 37°C and 5% CO_2_. The day after, the upper portion of the membrane contained in each well was cleaned to remove residual granulocytes and then the membranes were rinsed in Coomassie blue followed by distilled water. Each membrane was cut from the support and placed on a microscopy slide for counting.

### 2.3. Leukokit® Validation

The SOP for the validation includes the following quality control tests (QC):

- QC1 for the evaluation of hydrophobicity of ^99m^Tc-HMPAO
- QC2 for the evaluation of clumps after WBCs purification and labelling by visual inspection
- QC3 to calculate the labelling efficiency (LE) and labelling yield (LY) of WBCs
- QC4 to evaluate the sterility of the final product
- QC5 to evaluate the apyrogenicity of the final product
- QC6 to evaluate the vitality of radiolabelled cells by trypan blue exclusion test
- QC7 to evaluate the percentage of spontaneous release of ^99m^Tc-HMPAO from labelled WBCs at different time points

Leukokit® validation was performed in 6 patients who donated 60 ml of blood each (age 30–60), once given the written informed consent. For each patient, 60 ml of blood was withdrawn in two syringes with 6 ml of ACD each (30 ml and 30 ml of blood).

The first 36 ml was used for WBCs labelling with the “old Leukokit®” containing HES as the sedimentation agent; the other 36 ml was used for WBCs labelling with the “new Leukokit®” containing Gelofusine as the sedimentation agent.

The whole procedure requires between 2 h 45 min and 3 h 30 min depending on the erythrocyte-sedimentation rate (ESR) of the patient. Additional 4 h were necessary to complete all quality controls.

### 2.4. Clinical Analysis

Clinical comparison was performed between 80 patients (33 males; age 67.5 ± 14.2) injected with ^99m^Tc-HMPAO-WBCs, labelled using HES as the sedimentation agent, and 92 patients (38 males; age 68.2 ± 12.8) injected with ^99m^Tc-HMPAO-WBCs, labelled using Gelofusine as the sedimentation agent. Patients were affected by prosthetic joint infections, peripheral bone osteomyelitis, or vascular graft infection, as reported in Table 1.

Several parameters were considered: the radiolabelling efficiency (LE), final recovery yield (RY), and diagnostic outcome based on microbiology or 2-year follow-up.

For each group of patients, diagnostic accuracy, sensitivity, specificity, negative-predictive value (NPV), positive-predictive value (PPV), and their confidence intervals (CI) were calculated considering the number of patients as true positive (TP), true negative (TN), false positive (FP), and false negative (FN) based on the correspondence between the WBC scan and microbiology or follow-up.

### 2.5. Statistical Analysis

Comparisons of WBCs concentration, RBCs and PLTs contamination, and migration results were performed using Student's *t*-test for continuous variables after confirmation of normal distribution by the Kolmogorov–Smirnov test. Results in patients were statistically compared performing the Student's *t*-test, if normally distributed, otherwise performing the Mann–Whitney test.

All results were given as mean values ± SD or SE, unless otherwise indicated. Differences were considered significant when *p* values were <0.05. All calculations were performed using Prism 7 (GraphPad Software, La Jolla, CA, USA).

## 3. Results

### 3.1. WBC Isolation

**Figure 1:**
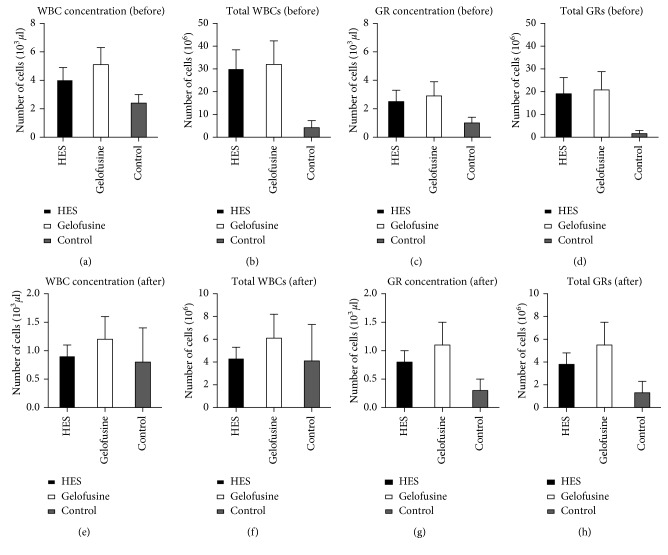
Recovery of total WBCs and granulocytes (GRs) when using HES, Gelofusine, or control (0.9% NaCl) before ((a)–(d)) and after ((e)–(h)) GR isolation (error bars = SE).

Gelofusine showed the best results in terms of number of recovered WBCs and granulocytes isolated from the blood of five healthy volunteers compared to HES and control (Figure 1 and Table 2). Differences are not statistically significant. Significant differences were observed when WBCs were purified from blood without any sedimentation agent, as expected, due to the low erythrocyte sedimentation speed (*p*=0.02 and *p*=0.03 for WBCs concentration (before), respectively, for HES and Gelofusine vs control; *p*=0.04 and *p*=0.07 for GRs concentration (before), respectively, for HES and Gelofusine vs control).

**Figure 2:**
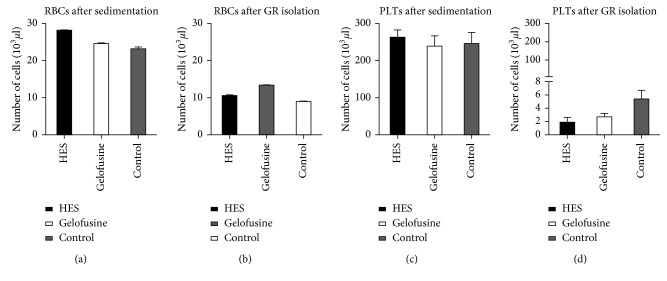
RBC and PLT contamination when using HES, Gelofusine, or control (0.9% NaCl) before and after GR isolation (error bars = SE).

On the contrary, the use of HES gave slightly lower RBCs and PLTs contamination (Figure 2). Differences are not statistically significant, except for PLTs contamination after GRs isolation between HES and control (*p*=0.04).

### 3.2. Vitality of Granulocytes

**Figure 3:**
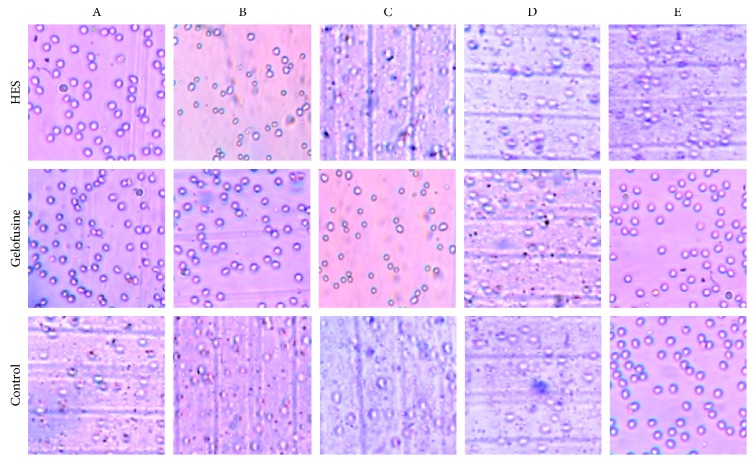
Trypan blue exclusion test of WBCs showing very high cell viability after erythrocyte sedimentation with HES, Gelofusine, or control solution. Each square represents a random field from 5 different subjects (A, B, C, D, and E).

High viability of isolated granulocytes was observed before and after purification, as revealed by the trypan blue exclusion test (Table 3 and Figure 3).

No statistical differences were observed between samples analyzed immediately after sedimentation (total leukocytes) or after centrifugation over the Lymphoprep® gradient (granulocytes). The same applies for samples sedimented with HES or Gelofusine or control.

### 3.3. Granulocyte Migration Assay

**Figure 4:**
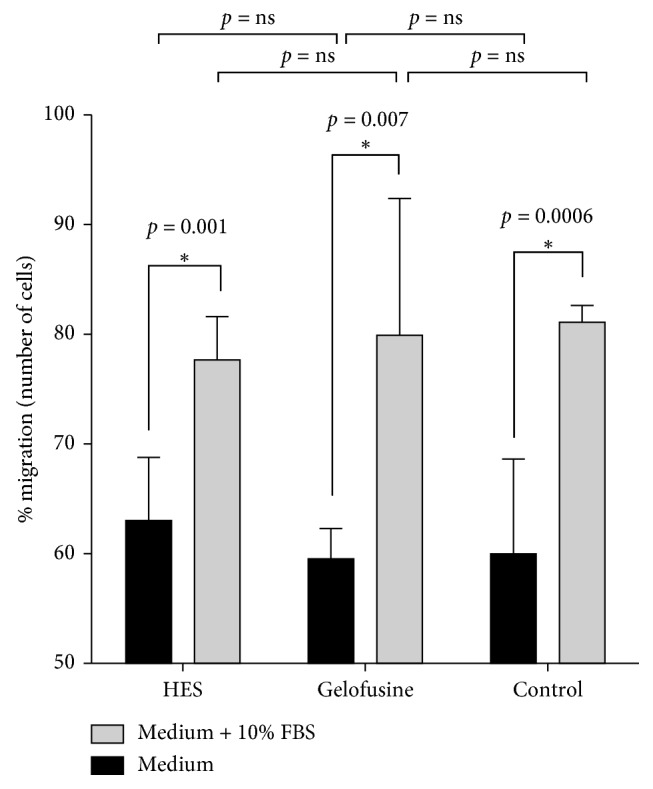
Granulocyte migration assay performed using medium with (grey bars) or without (black bars) 10% FBS (error bars = SD).

**Figure 5:**
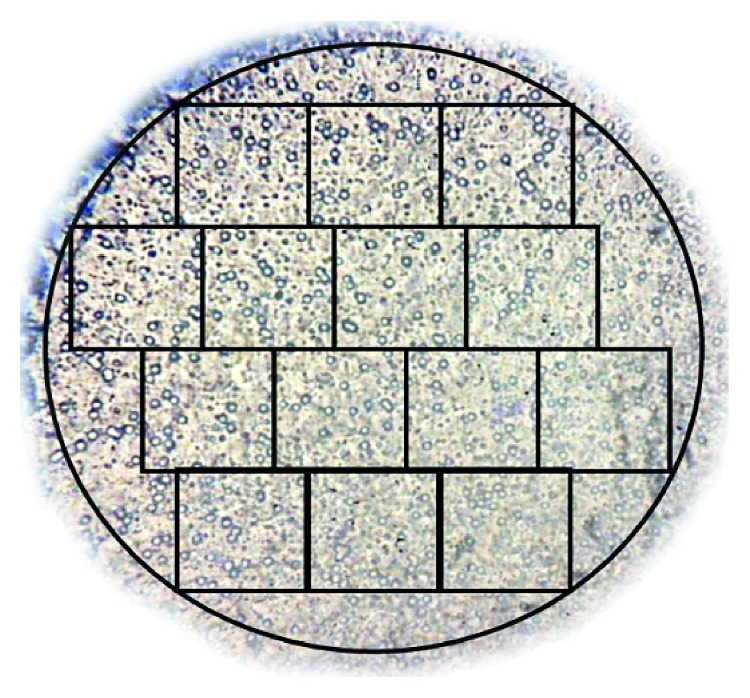
Random field of Transwell membrane from subject C.

Isolated granulocytes retained their ability to migrate in response to attracting stimuli, as revealed by migration assays performed in medium with or without 10% FBS (Figures 4 and 5). There was a significant difference between groups with or without FBS stimulation (*p*=0.001, *p*=0.007, and *p*=0.0006 for HES, Gelofusine, or control groups, respectively).

In addition, there was no significant difference between cells prepared with HES or Gelofusine or without any sedimentation agent as control.

### 3.4. Leukokit® Validation

The main QCs that are reported here (and of interest for the comparison of Gelofusine vs HES) are the labelling efficiency (LE), the labelling yield (LY), the vitality of labelled cells using the trypan blue exclusion test, and the spontaneous *in vitro* release of ^99m^Tc-HMPAO from labelled WBCs at different time points incubated at 37°C (10′, 1 h, and 4 h).

The CRP volume was different for each patient, depending on ESR of each one (range 20–30 ml).

**Figure 6:**
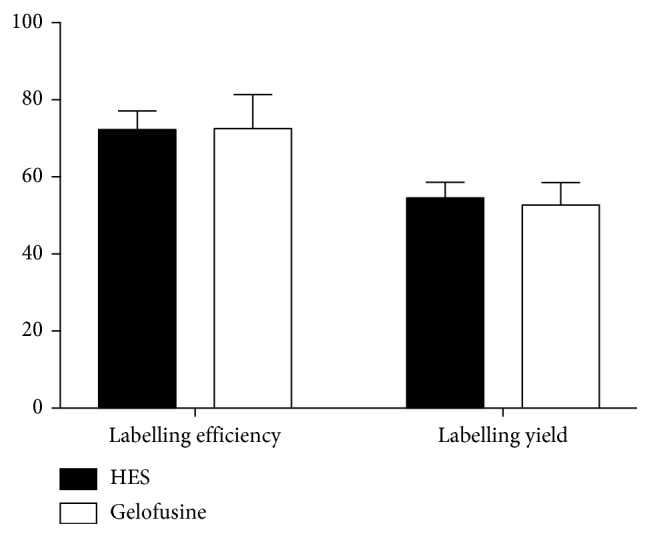
Graphic representation of labelling efficiency and labelling yield with different kits (data are mean of 6 subjects ± SD). The vitality test showed the same result for both sedimentation agents with a mean value ± SD equal to 99.7 ± 0.4%.

The average labelling efficiency (LE) was similar between the two sedimentation agents: 72.3 ± 4.8% for HES and 72.5 ± 8.9% for Gelofusine; the labelling yield (LY) was slightly better for HES (54.5 ± 4.1%) than Gelofusine (52.7 ± 5.8%). Differences were not statistically significant (Figure 6).

**Figure 7:**
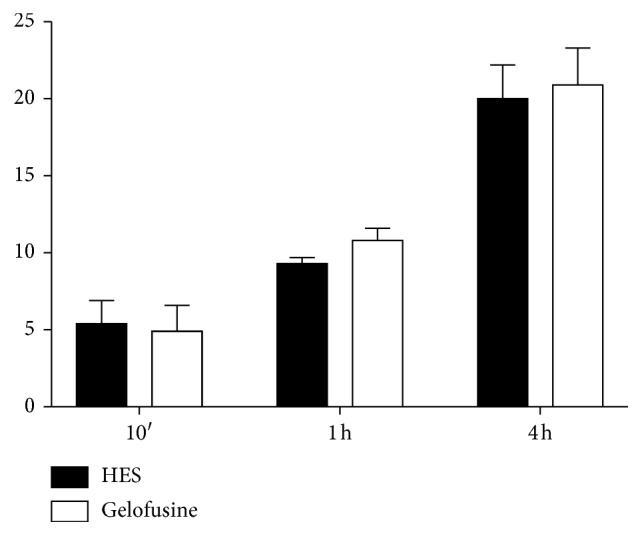
Graphic representation of the spontaneous *in vitro* release of ^99m^Tc-HMPAO from labelled WBCs at different time points (data are mean of 6 subjects ± SD).

Finally, the release of ^99m^Tc-HMPAO from labelled WBCs was evaluated. Results showed a less release from cells at 10 minutes for Gelofusine (4.9 ± 1.7%) in comparison to HES (5.4 ± 1.5%), showing similar results at 1 h and 4 h (10.8 ± 0.8% vs 9.3 ± 0.4%, respectively, at 1 h and 20.9 ± 2.4% vs 20 ± 2.2%, respectively, at 4 h) (Figure 7). All differences are not statistically significant.

### 3.5. Clinical Analysis

For the “new Leukokit®,” the LE and RY, calculated on 92 samples, were 71.4 ± 11.4% and 55.6 ± 9.4%, respectively, whereas for the “old Leukokit®”, the LE and RY, calculated on 80 samples, were 74.5 ± 9.6% and 54.8 ± 10.4%, respectively. Both differences were not statistically significant (*t*-test *p*=0.06 and *p*=0.57, respectively, for LE and RY).

As far as the diagnostic performance of the two kits is concerned, we were able to include only 58 patients for the “new Leukokit®” and 44 patients for the “old Leukokit®” because of the availability of reliable microbiological results or clinical data during the 2-year follow-up.

As shown in Table 1, there were no statistically significant differences between the two groups of patients either if we consider them in all or by single pathology (Pearson's chi-square test).

## 4. Discussion

WBCs isolation and radiolabelling are critical steps to obtain an available radiopharmaceutical with high purity and labelling efficiency, suitable for WBCs scintigraphy [13–16]. The availability of a sterile device, Leukokit®, has absolutely provided an instrument to facilitate the whole procedure, reducing time and assuring sterility as reported in the recent guidelines published by EANM Committee [14]. The utility and safety of Leukokit® were reported in several studies that obtained high values of LE and RY comparable to our study [3, 8–11, 17, 18]. These studies used ^99m^Tc-HMPAO-WBCs with Leukokit®. Hence, Leukokit® has been used for WBC labelling also using other chelating agents for ^99m^Tc [19] or other isotopes such as ^111^In [20, 21], ^18^F-FDG [22, 23], and ^64^CuCl [24]. Thus, the use of Leukokit® plays a pivotal role for WBCs isolation and radiolabelling procedure in clinical practice. Indeed, Gelofusine was chosen as an alternative to HES as plasma expander within the Leukokit®. It is commercially available at 4% concentration with a molecular weight average of 26500 Da.

In our study, HES and Gelofusine were compared *in vitro* and in clinical practice after the introduction of Gelofusine in the Leukokit®.

From our results, Gelofusine allowed a better separation of granulocytes from whole blood of healthy subjects as compared to HES, with optimal cell vitality.

From data collected in patients, labelling efficiency and labelling yield were similar for the two kits, and diagnostic accuracy, sensitivity, specificity, PPV, and NPV were also not significantly different for both sedimentation agents. These results are in agreement with data previously published [15, 16], being the overall diagnostic accuracy of the two tests equal to 98.3% and 90.9% (for Gelofusine and HES, respectively). In this study, we included only patients with a clearly defined diagnosis, mainly because of availability of microbiological data obtained during surgery or more rarely because of a 2-year follow-up without use of any antibiotic therapy. This selection may be at risk of bias, but rather than providing data in support of WBCs scan, we aimed at comparing with the same methodology and same source of bias two different groups of patients. When subdividing patients for different pathologies, we noticed that the number of patients with suspected vascular graft infection is too few to draw any conclusion, but even if removed from total, the overall results are the same with no statistical difference between WBCs scans in patients using “old Leukokit®” and “new Leukokit®”. In particular, in knee prosthesis, we found a sensitivity and specificity of 100% and 94.4%, respectively, for the “old Leukokit®” and a sensitivity and specificity of 100% and 100%, respectively, for the “new Leukokit®”. Similarly, in hip prosthesis, we found a sensitivity and specificity of 100% and 83.3%, respectively, for the “old Leukokit®” and a sensitivity and specificity of 66.7% and 100%, respectively, for the “new Leukokit®”. These results are well in agreement with those recently published in two meta-analysis by Verberne et al. in which they report an overall sensitivity and specificity for radiolabelled WBCs scintigraphy in knee prosthesis of 88% and 77%, respectively [15], and in hip prosthesis a sensitivity and specificity of 88% and 92%, respectively [16], although in all mentioned studies WBCs were labelled without the use of Leukokit®.

A possible criticism to our work can be raised by the consideration that we used blood of normal subjects for the *in vitro* experiments and not from patients. Indeed, the low ESR in normal subjects could have negatively influenced the sedimentation of RBCs and the purification of WBCs from RBCs and PLTs. However, the choice of using blood from normal subjects was done on purpose for ethical reasons and for evaluating the efficacy of Gelofusine and HES in the worst situation (i.e., when ESR is very low).

## 5. Conclusion

Both HES and Gelofusine sedimentation agents allowed reproducible separation of granulocytes from whole blood with a high percentage of purity and vitality as required by EANM guidelines.

In particular, Gelofusine can be considered a suitable alternative of HES for WBCs separation and labelling, yielding to high labelling efficiency, without cell damage and high diagnostic accuracy.

## Figures and Tables

**Table 1:** Summary of clinical results of patients with WBCs prepared using HES-Leukokit® or Gelofusine-Leukokit®.

		Accuracy (%)	Sensitivity (%)	Specificity (%)	PPV (%)	NPV (%)
HES	Osteomyelitis (*n*=8)	87.5	50	100	100	85.7
	Hip prosthesis (*n*=13)	84.6	100	83.3	33.3	100
	Vascular grafts (*n*=3)	100	100	100	100	100
	Knee prosthesis (*n*=20)	95	100	94.4	66.7	100
	All (*n*=44)	90.9 (74.9–96.1)	83.3 (22.3–95.7)	92.1 (78.1–98.3)	62.5 (18.4–90.1)	97.2 (81.3–99.3)


Gelofusine	Osteomyelitis (*n*=16)	100	100	100	100	100
	Hip prosthesis (*n*=16)	93.8	66.7	100	100	92.9
	Vascular grafts (*n*=5)	100	100	100	100	100
	Knee prosthesis (*n*=21)	100	100	100	100	100
	All (*n*=58)	98.3 (90.8–100)	91.7 (61.5–99.8)	100 (92.3–100)	100 (71.5–100)	97.9 (88.7–99.9)

All values are expressed as percentage, and the confidence intervals are given in brackets.

**Table 2:** Values of recovered blood elements after erythrocyte sedimentation with HES, Gelofusine, or control (before). The analysis was repeated after granulocyte purification by centrifugation.

	HES		Gelofusine		Control	
	Before	After	Before	After	Before	After
Mean WBCs (10^6^)	29.9 ± 8.5	4.3 ± 1.0	32.0 ± 10.3	6.1 ± 2.1	4.2 ± 3.1	4.1 ± 3.2
Mean GRs (10^6^)	19.1 ± 7.1	3.8 ± 1.0	20.8 ± 8.0	5.5 ± 2.0	1.6 ± 1.4	1.3 ± 1.0
Mean RBCs (10^6^)	0.2 ± 0.04	0.1 ± 0.01	0.1 ± 0.04	0.1 ± 0.01	0.02 ± 0.02	0.05 ± 0.02
Mean PLTs (10^6^)	1893.4 ± 251.3	9.7 ± 3.6	1450.3 ± 246.8	13.5 ± 2.7	304.5 ± 170.4	27.2 ± 6.3

**Table 3:** Viability of mixed leukocytes or purified granulocytes tested by the trypan blue exclusion test.

	HES		Gelofusine		Control	
Subject	Leukocytes (%)	Granulocytes (%)	Leukocytes (%)	Granulocytes (%)	Leukocytes (%)	Granulocytes (%)
A	99.8	99.2	99.9	99.4	98.8	98.5
B	99.2	99.1	99.7	99.5	99.6	99.3
C	99.4	99.5	99.4	99.2	99.5	99.5
D	99.3	99.6	99.9	99.3	99.6	99.8
E	99	99.5	99.1	99.8	99.2	99.4

## Data Availability

The data used to support the findings of this study are available from the corresponding author upon request.
